# Effects of Dietary Curcumin Supplementation Under High-Level Fishmeal Replacement with *Clostridium autoethanogenum* Protein in *Penaeus vannamei*: Growth Performance, Immunity, Intestinal Health, and Transcriptome Responses

**DOI:** 10.3390/biology15161353

**Published:** 2026-08-10

**Authors:** Yue Yin, Sheng Ke, Jian Chen, Ling Pan, Yudong Zheng, Hongming Wang, Lili Shi, Shuang Zhang

**Affiliations:** 1 College of Fisheries, Guangdong Ocean University, Zhanjiang 524088, China; m15704581120_1@163.com (Y.Y.); 17850140976@163.com (S.K.); chenjian@catas.cn (J.C.); lingpan0304@catas.cn (L.P.); yd_zheng96@126.com (Y.Z.); wanghongming97@163.com (H.W.); 2 Aquatic Animals Precision Nutrition and High Efficiency Feed Engineering Research Center of Guangdong Province, Zhanjiang 524088, China; 3 Zhanjiang Experimental Station, Chinese Academy of Tropical Agricultural Sciences, Zhanjiang 524088, China

**Keywords:** *Penaeus vannamei*, curcumin, *Clostridium autoethanogenum* protein, growth performance, intestinal health, transcriptome responses

## Abstract

*Clostridium autoethanogenum* protein (CAP) offers a sustainable fishmeal alternative, but high inclusion may impair shrimp growth and health. This study evaluated the responses associated with dietary curcumin (CUR) supplementation in *Penaeus vannamei* fed a diet in which 50% of the fishmeal was replaced with CAP. Three diets were formulated: a fishmeal-based control diet (FM), a low-fishmeal diet in which 50% of the fishmeal was replaced with CAP (CAP), and the CAP diet supplemented with 60 mg/kg CUR (CTER). After an 8-week feeding trial, *P. vannamei* fed the CAP diet exhibited significantly lower final body weight, weight gain rate, and specific growth rate, accompanied by adverse histological changes in the intestine and hepatopancreas, reduced hepatopancreatic immune enzyme activities, and alterations in the intestinal microbiota. Compared with the CAP group, CUR supplementation was associated with improved growth performance, higher survival following WSSV exposure, improved hepatopancreatic and intestinal morphology, increased immune enzyme activities, greater microbial richness, and shifts in the relative abundance of several dominant bacterial taxa. Transcriptomic analysis further suggested that the beneficial effects of CUR may be partly associated with differential expression of genes enriched in metabolism- and immunity-related pathways. Overall, dietary CUR supplementation was associated with broad improvements in growth, tissue condition, nonspecific immune responses, intestinal microbial profiles, and post-challenge survival under the high-CAP dietary condition. These findings support the potential application of CUR as a functional additive in CAP-based, low-fishmeal diets for *P. vannamei*.

## Simple Summary

Fishmeal is an important but costly and limited protein ingredient in shrimp feed. *Clostridium autoethanogenum* protein is a promising alternative, yet high-level inclusion levels may adversely affect shrimp growth and health. This study evaluated whether dietary curcumin supplementation in *Penaeus vannamei* fed a diet in which 50% of the fishmeal was replaced with CAP. Three experimental groups were designed in this study: a fishmeal control, a high-CAP diet, and the same high-CAP diet supplemented with curcumin. After eight weeks, shrimp fed the high-CAP diet showed reduced growth, adverse histological changes in the intestine and hepatopancreas, lower nonspecific immune enzyme activities, and alterations in intestinal microbial community composition. Compared with the high-CAP group, curcumin supplementation was associated with improvements in growth performance, tissue morphology, immune enzyme activities, and intestinal microbial profiles. Transcriptomic analysis suggested that identified changes were associated with metabolic and immune-related biological processes. Because a fishmeal-based diet supplemented with curcumin was not included, these findings should be interpreted specifically under high-CAP dietary conditions. Overall, the results support the potential application of curcumin as a functional feed additive in CAP-based, low-fishmeal diets for shrimp.

## 1. Introduction

The edible tissue of *Penaeus vannamei* is characterized by a high protein content, approximately 20–22%, and a low lipid content, less than 1%, making it a nutritious and healthy seafood product [1,2]. Owing to its rapid growth, high survival rate under intensive culture conditions, broad salinity tolerance, and strong environmental adaptability, *P. vannamei* has become the dominant species in modern shrimp aquaculture, accounting for approximately 80% of global farmed shrimp production. In shrimp aquaculture, feed quality is a critical factor determining production efficiency and economic profitability. With the continuous expansion of global shrimp farming, the development of nutritionally balanced, cost-effective, and sustainable feed formulations is essential for maintaining industrial productivity and promoting the long-term sustainability of the aquaculture industry.

*P. vannamei* is an omnivorous species with a relatively high dietary protein requirement; therefore, dietary protein is a key nutritional factor affecting shrimp growth performance and feed utilization efficiency [3]. Fishmeal has long been regarded as an ideal protein source for *P. vannamei* because of its balanced amino acid profile, high digestibility, good palatability, and abundance of functional components, including taurine, nucleotides, and bioactive peptides [4,5,6]. The global aquaculture industry consumes approximately 4 million tonnes of fishmeal annually, of which about 25% is used in shrimp farming [7]. However, the limited availability of fishmeal resources, rising prices, and increasing ecological pressure have made the reduction of fishmeal inclusion in aquafeeds an important requirement for the sustainable development of the industry [8,9]. Therefore, identifying sustainable protein sources that can replace fishmeal without compromising shrimp growth, health, and immune function has become a major focus in aquatic nutrition research.

Among various alternative protein sources, single-cell protein (SCP) has attracted considerable attention because of its high protein content, stable production, appropriate amino acid profile, and independence from arable land and marine resources [10,11]. Recent studies have expanded the application of SCP in shrimp nutrition, showing that microalgal SCP derived from *Chlorella sorokiniana* and methanotrophic bacterial protein can support growth, intestinal microbial homeostasis, antioxidant responses, and disease resistance in *P. vannamei* when used at appropriate dietary levels [12,13]. *Clostridium autoethanogenum* protein (CAP) is a novel single-cell protein produced from industrial waste gases through gas fermentation. It is characterized by high crude protein content, good digestibility, and the absence of common anti-nutritional factors such as trypsin inhibitors and phytic acid [14,15,16,17]. Previous studies have shown that moderate replacement of fishmeal with CAP can maintain or even improve growth performance, antioxidant capacity, and intestinal health in some aquatic animals [18,19]. In *P. vannamei*, approximately 30% fishmeal replacement by CAP has been reported to maintain growth performance, whereas higher replacement levels (≥45%) may adversely affect growth and related physiological responses [20,21]. More recently, CAP replacement of up to 60% was reported to maintain shrimp growth under a commercial-type dietary formulation [22]. Accordingly, 50% fishmeal replacement was selected in the present study as a moderately high CAP challenge level, exceeding the generally well-tolerated level while avoiding the potentially more severe effects associated with very high replacement levels (70–100%). At high inclusion levels, CAP may not fully reproduce the nutritional and functional value of fishmeal because of potential mismatches in limiting essential amino acids and digestible energy, as well as the relatively high nucleic-acid fraction and distinct cell-wall components of bacterial biomass. Moreover, extensive fishmeal replacement reduces dietary palatability factors and functional nutrients, including taurine, nucleotides, phospholipids, and bioactive peptides, which may collectively impair feed intake, nutrient utilization, intestinal health, and growth [23,24,25]. In *P. vannamei*, high-level replacement of fishmeal with CAP has been shown to reduce growth performance and adversely affect hepatopancreatic and intestinal health [26]. These findings suggest that, under high-CAP dietary conditions, functional nutritional regulation strategies are needed to alleviate the potential negative effects associated with CAP inclusion.

In recent years, functional feed additives, particularly plant-derived bioactive compounds, have received increasing attention in aquaculture. For example, *Lycium barbarum* polysaccharides can enhance antioxidant capacity and immune function in *Macrobrachium rosenbergii* under ammonia nitrogen stress [27], whereas daidzein can alleviate alkaline stress-induced liver injury in common carp [28]. Curcumin (CUR) is a natural polyphenolic compound extracted from turmeric and possesses strong antioxidant, anti-inflammatory, immunomodulatory, and intestinal protective properties. The phenolic hydroxyl groups and β-diketone structure in its molecule confer strong free radical-scavenging capacity and biological activity [29]. Previous studies have demonstrated that dietary CUR supplementation can improve multiple physiological functions in aquatic animals. In Nile tilapia, dietary CUR supplementation enhanced growth performance and antioxidant capacity [30]. In gilthead seabream, dietary CUR improved digestive function [31]. In addition, CUR supplementation markedly improved intestinal health in crucian carp and juvenile grass carp [32,33]. In *P. vannamei*, curcumin-related preparations have also been shown to improve growth performance, enhance antioxidant status, and strengthen immune defense capacity [34,35]. Additional studies in *P. vannamei* have shown that curcumin-loaded chitosan nanoparticles can enhance growth and nonspecific immune responses, including under *Vibrio harveyi* challenge, while dietary curcumin preparations have also been investigated in shrimp exposed to salinity stress [36,37]. More recently, the application of CUR in low-fishmeal and fishmeal-replacement diets has attracted increasing interest. Studies in largemouth bass and *M. rosenbergii* have shown that appropriate CUR supplementation can alleviate growth depression, reduced feed utilization, enhanced oxidative stress, suppressed immune function, and intestinal structural damage induced by low-fishmeal or high alternative-protein diets [38,39]. Nevertheless, these studies involved alternative protein sources other than CAP or aquatic species other than *P. vannamei*, and the effects of CUR under high-CAP dietary conditions in shrimp remain poorly understood.

On this basis, the present study aimed to evaluate whether dietary CUR supplementation could alleviate the adverse effects of replacing 50% of fishmeal with CAP in *P. vannamei*. To this end, growth performance, feed utilization efficiency, immune parameters, intestinal and hepatopancreatic histology, intestinal microbiota composition, and hepatopancreatic transcriptomic responses were systematically analyzed. We hypothesized that high CAP inclusion would adversely affect nutrient utilization, intestinal and hepatopancreatic integrity, innate immune status, and microbial homeostasis, whereas dietary CUR supplementation would partially alleviate these responses. This study could provide a theoretical basis for the development of sustainable low-fishmeal diets for *P. vannamei*.

## 2. Materials and Methods

### 2.1. Preparation of Experimental Diets

CUR (≥95%) was purchased from Guangzhou Kehu Biotechnology Co., Ltd. (Guangzhou, China). Three approximately isonitrogenous and isolipidic experimental diets were formulated: a fishmeal-based control diet (FM), a diet in which 50% of the fishmeal was replaced with CAP (CAP), and the CAP diet supplemented with 60 mg/kg CUR (CTER). The CUR supplementation level of 60 mg/kg (the nominal inclusion level) was selected based on previous studies in *P. vannamei* and our preliminary dose–response observations under conventional dietary conditions [34,35,36,37,40]. The present experiment was designed specifically to evaluate whether CUR supplementation could alleviate the adverse effects associated with high-level fishmeal replacement by CAP. Red fishmeal was used as the primary protein source, together with soybean meal, shrimp shell meal, peanut meal, and flour. For preparation of the CTER diet, CUR was accurately weighed and completely dissolved in the lipid mixture consisting of fish oil, soybean oil, and soy lecithin. All dry ingredients were first pulverized, passed through an 80-mesh sieve, accurately weighed according to the diet formulation, and thoroughly mixed in a V-type vertical mixer (Zhanjiang, China). The mixed dry ingredients were subsequently passed through an 80-mesh sieve for a second time to ensure the homogeneity of the dry powder matrix. After the second sieving step, the lipid mixture containing dissolved CUR was gradually added to the homogenized dry ingredients, and the mixture was thoroughly kneaded until a uniform appearance and consistency were achieved. The FM and CAP diets were prepared following the same procedure, with the corresponding lipid mixture added after the second sieving step. The diets were pelleted using a twin-screw extruder (F-26, South China University of Technology, Guangzhou, China). Following extrusion, the pellets were post-conditioned at 90 °C for 15 min and subsequently air-dried at 26 °C for 48 h. The dried pellets were transferred into sealed bags, labeled, and stored at −20 °C until use. The formulation and proximate composition of the experimental diets are presented in Table 1.

### 2.2. Feeding Trial

Healthy *P. vannamei* (initial body weight: 0.40 ± 0.02 g) obtained from Guangdong Zhanjiang Yuehai Aquatic Seedling Co., Ltd. (Zhanjiaing, China) were randomly assigned to three dietary groups with three replicates each (40 shrimp per 300 L PVC tank). Each tank was considered an independent experimental unit. The 8-week trial was conducted in an indoor aquaculture workshop. Shrimp were fed four times daily (07:00, 11:00, 17:00, 21:00) at an initial ration of 8–9% of total biomass, and then adjusted to apparent satiation (defined as a marked decline in feeding activity with a small amount of uneaten feed remaining). Water quality parameters were maintained as follows: temperature, 27 ± 3 °C; salinity, 28 ± 5‰; dissolved oxygen, ≥5.0 mg/L; and pH, 7.5–8.5.

### 2.3. Sample Collection

At the end of the 8-week feeding trial, all shrimp were fasted for 24 h. The total number and total weight of shrimp in each tank were then recorded for the calculation of growth performance. Each dietary treatment consisted of three independent tanks, and each tank was considered one biological replicate and the experimental unit. Subsequently, 13 shrimp were randomly selected from each tank (from apparently healthy individuals, without deliberate size or sex selection). For histological analysis, the hepatopancreas and intestine from one shrimp in each tank were dissected and fixed, resulting in three independent biological replicates per dietary treatment. For the other tissue-based analyses, tissues collected from three shrimp within the same tank were pooled to generate one composite sample representing that tank. The hepatopancreas from three shrimp were collected for nonspecific immune enzyme activity assays. The intestines from another three shrimp were collected for digestive enzyme activity assays. In addition, the intestines from another three shrimp were collected for intestinal microbiota analysis. Finally, the hepatopancreas from the remaining three shrimp were collected for transcriptomic analysis. The hepatopancreatic and intestinal samples used for enzyme activity assays, intestinal microbiota analysis, and transcriptomic analysis were placed in RNase-free tubes, immediately frozen in liquid nitrogen, and stored at −80 °C until analysis. For histological analysis, the hepatopancreatic and intestinal tissues were carefully dissected and immediately fixed in Bouin’s solution for 24 h.

### 2.4. Growth Performance Analysis

The number and total weight of shrimp in each tank were recorded to calculate survival rate (SR), weight gain rate (WGR), specific growth rate (SGR), and feed conversion ratio (FCR). The growth indicators were determined using the recorded data and the following formulas:

WGR (%) = (Final body weight − Initial body weight)/Initial body weight × 100

SGR (%/d) = [ln (Final body weight) − ln (Initial body weight)]/days × 100

SR (%) = Final shrimp number/Initial shrimp number × 100

FCR = Feed intake/(Final body weight − Initial body weight)

### 2.5. Challenge Test with White Spot Syndrome Virus (WSSV)

Frozen tissues from WSSV-infected shrimp stored at −80 °C were minced, homogenized, centrifuged, and filtered to obtain a crude viral extract. Healthy shrimp were then intramuscularly injected with 50 μL of the extract for viral amplification. Within 24 h post-injection, muscle tissue from infected shrimp was collected and processed using the same procedure to prepare the challenge inoculum. For the formal challenge, the WSSV inoculum was adjusted to 1 × 10^6^ copies/μL. At the end of the feeding trial, shrimp from the three replicate tanks within each dietary treatment were pooled, and 20 apparently healthy shrimp of similar body size were randomly selected from each pooled group. The selected shrimp were maintained in one challenge tank per dietary treatment, with no independent tank-level replication during the challenge phase. Each shrimp was intramuscularly injected with 10 μL of the WSSV suspension between the third and fourth abdominal segments. No feed was provided during the challenge period. Mortality was recorded at 4-h intervals, and dead shrimp were removed. The challenge lasted 72 h.

### 2.6. Histology Analysis

Hepatopancreas and intestinal tissues were fixed in Bouin’s solution for 24 h. After fixation, the tissue samples were dehydrated through a graded ethanol series for 20 min at each concentration, cleared in xylene, infiltrated with molten paraffin, and embedded in paraffin using a standard tissue-processing protocol. The paraffin-embedded tissues were sectioned and mounted on glass slides. The sections were subsequently deparaffinized, rehydrated through a descending ethanol series, and stained with hematoxylin and eosin (H&E) to visualize cellular and morphological structures. After staining, the sections were dehydrated, cleared, mounted with a permanent mounting medium, and sealed for long-term preservation. The stained sections were examined and photographed using a light microscope (Nikon ECLIPSE Ni-E, Nikon, Minato-ku, Japan). Intestinal epithelial cell height (EC), fold height (FH), and muscle layer thickness (MT) were measured using ImageJ software (version 2.1.0). For each tank, ten randomly selected positions within one intestinal section were measured. These measurements were treated as technical subsamples and averaged to obtain one representative value for that tank. Thus, three tank-level values were used for statistical analysis in each dietary treatment.

### 2.7. Assessment of Non-Specific Immune Indicators and Digestive Enzyme Activities

Hepatopancreas samples and intestinal tissues were retrieved from storage at −80 °C and thawed on ice. Hepatopancreas and intestinal tissues were separately homogenized in ice-cold 0.9% saline at a tissue-to-saline ratio of 1:9 (*w*/*v*). The homogenates were centrifuged at 4000 rpm for 15 min at 4 °C, and the resulting supernatants were collected. The hepatopancreas supernatants were used to measure the activities of PO (YJ254195), alkaline phosphatase (AKP, YJ981141), and acid phosphatase (ACP, YJ362941). The intestinal supernatants were analyzed for amylase (YJ665236), protease (YJ652354), and lipase (YJ360195) activities. The soluble protein concentration of each tissue supernatant was determined using a BCA protein assay kit (BCAP-W96-N (1620), Shanghai China) according to the manufacturer’s instructions. Briefly, protein concentration was quantified based on the formation of a purple BCA–Cu^+^ complex, and absorbance was measured at 562 nm. The activities of PO, AKP, ACP, amylase, protease, and lipase were normalized to the protein content of the corresponding tissue supernatant and expressed as U/g prot.

### 2.8. Intestinal Microflora

Total bacterial DNA was extracted from intestinal samples using the HiPure Soil DNA Kit (Guangzhou Magen Co., Ltd., Guangzhou China). The V3–4 hypervariable region of the bacterial 16S rRNA gene was amplified using the primers 341F (CCTACGGG-NGGCWGCAG) and 806R (GGACTACHVGGGTATCTAAT). Following DNA purification and concentration measurement, sequencing libraries were prepared and analyzed by Guangzhou Genedenovo Biotechnology Co., Ltd. (Guangzhou, China) using the Illumina HiSeq 2500 platform with 250-bp paired-end sequencing. The raw reads were processed with FASTP v0.18.0 to remove low-quality sequences. Subsequently, paired-end reads were assembled using FLASH (version 1.2.11), and the merged sequences were further screened to generate high-quality clean tags. UPARSE v9.2.64 was then used to cluster the sequences into operational taxonomic units at 97% sequence similarity, with chimeric sequences excluded during the process. Representative sequences from each OTU were taxonomically classified using the RDP Classifier v2.2 against the SILVA database release 138.1, with a bootstrap confidence threshold of 0.80. QIIME [41,42] was utilized to compute several indices, such as OTUs, ACE, Simpson, and Goods coverage, in order to evaluate alpha diversity. Beta-diversity patterns were visualized by principal coordinate analysis (PCoA) based on Bray–Curtis dissimilarities. Taxonomic composition at the phylum and genus levels was summarized and presented as relative abundance. Because of the compositional nature of the data and the limited number of biological replicates, taxonomic relative-abundance profiles were interpreted descriptively. Microbial functional profiles were predicted using PICRUSt2 (version 2.5.2), and were interpreted as predicted functional potentials rather than experimentally verified microbial activities. The sequencing data were deposited in the NCBI database under BioProject accession number PRJNA1497136.

### 2.9. Transcriptome Sequencing Analysis

Total RNA was extracted from hepatopancreas samples using TRIzol reagent (Invitrogen, Waltham, MA, USA). RNA purity, concentration, and integrity were assessed using a NanoDrop spectrophotometer (NanoDrop 2000, Thermo Fisher Scientific, Waltham, MA, USA) and RNase-free 1% agarose gel electrophoresis. The samples with RNA integrity numbers (RIN) ≥ 7 were used for library construction. After the RNA samples met the required quality criteria, library construction and sequencing were performed by Guangzhou Genedenovo Biotechnology Co., Ltd. (Guangzhou, China). Paired-end sequencing was conducted on the Illumina HiSeq 2000 platform to generate approximately 200-bp paired-end reads. Raw reads were filtered using fastp to remove adapter-containing reads, reads containing more than 10% unknown bases, and low-quality reads. The resulting clean reads were aligned to the *P. vannamei* reference genome (NCBI Accession No. PRJNA438564) using HISAT2 version 2.2.4, and transcripts were reconstructed and merged using StringTie version 1.3.1. Differential gene expression analysis was performed using DESeq2 version 1.42.0, with gene counts normalized using the median-of-ratios method. Principal component analysis and sample-correlation analysis were conducted using normalized expression data to evaluate biological replicate reproducibility and potential batch effects. Comparisons were defined as FM vs. CAP and CAP vs. CTER. Positive log2 fold-change values (|log2FC|) indicate higher expression in the second group relative to the first group. To account for multiple comparisons, raw *p*-values were adjusted using the Benjamini–Hochberg procedure to control the false discovery rate (FDR). Genes with an adjusted *p*-value (FDR) < 0.05 and an absolute |log2FC| ≥ 1.5 were considered differentially expressed genes (DEGs). Gene Ontology (GO) and Kyoto Encyclopedia of Genes and Genomes (KEGG) enrichment analyses were subsequently performed to determine the biological functions and signaling pathways associated with the DEGs. *p*-values generated from the enrichment analyses were also corrected for multiple testing using the Benjamini–Hochberg procedure, and GO terms or KEGG pathways with an adjusted *p*-value (FDR) < 0.05 were considered significantly enriched. The sequencing data were deposited in the NCBI database under BioProject accession number PRJNA1107795.

### 2.10. RT-qPCR Analyses of Gene Expression

Total RNA isolated from hepatopancreas tissue (TransGen, Beijing, China) was reverse transcribed into cDNA using a reverse transcription kit (TaKaRa, Kusatsu, Shiga, Japan). qPCR was performed with SYBR Green (Roche Applied Science, Mannheim, Germany) under the following cycling conditions: initial denaturation at 95 °C for 300 s; 45 amplification cycles at 95 °C for 10 s, 58 °C for 15 s, and 72 °C for 15 s; melting curve analysis at 95 °C for 10 s, 65 °C for 60 s, and 97 °C for 1 s, followed by annealing at 37 °C for 30 s. Relative quantification of gene expression was conducted using the 2^−ΔΔCt^ method, with the geometric mean of elongation factor-1 alpha (*EF-1α*) serving as the endogenous reference for data normalization. The correlation between the RNA-seq and qPCR results was evaluated by linear regression analysis. In order to validate the transcriptomic data, nine differentially expressed genes (DEGs) were selected at random, and their relative expression levels were examined by qPCR using the primers listed in Table 2.

### 2.11. Statistical Analysis

Statistical analyses were performed using SPSS version 26.0 for Windows (IBM Corp., Chicago, IL, USA). The tank was considered the experimental unit for growth performance and other treatment-related variables. Before one-way analysis of variance (ANOVA), data were tested for normality using the Shapiro–Wilk test and for homogeneity of variance using Levene’s test. When the assumptions of normality and homogeneity of variance were satisfied, differences among dietary treatments were analyzed by one-way ANOVA followed by Duncan’s multiple range test. When these assumptions were not satisfied, the data were appropriately transformed and retested. If the transformed data still failed to meet the assumptions of parametric analysis, the Kruskal–Wallis non-parametric test was applied, followed by an appropriate post hoc multiple-comparison test. Data are presented as the mean ± standard deviation (SD), and differences were considered statistically significant at *p* < 0.05. Survival differences among groups following the WSSV challenge were analyzed by the Mantel–Cox method (log-rank χ^2^ test) using GraphPad Prism 10.5.0 software.

## 3. Results

### 3.1. Growth Performance

Compared with the FM group, the CAP group showed significantly lower FBW, WGR, and SGR (*p* < 0.05). After CUR supplementation, these parameters in the CTER group were significantly higher than those in the CAP group (*p* < 0.05) and did not differ significantly from those in the FM group (*p* > 0.05; Table 3).

### 3.2. Resistance to WSSV

**Figure 1:**
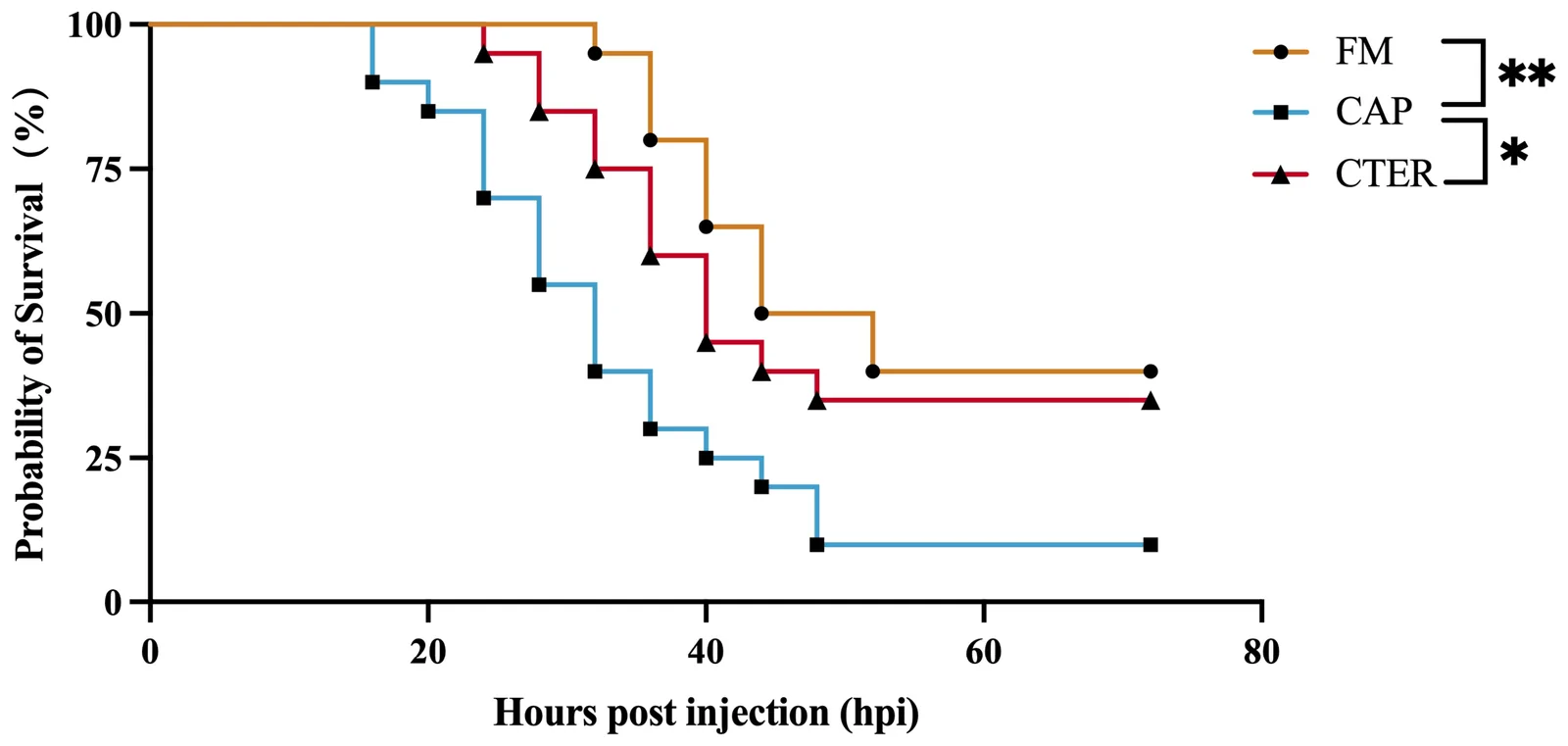
Survival of *P.vannamei* after WSSV infection. No asterisk represents no significant difference (*p* > 0.05); an asterisk represents a significant difference (* represents *p* < 0.05; ** represents *p* < 0.01).

Dietary inclusion of CUR in the high-CAP replacement FM diets significantly influenced the SR of *P. vannamei* following WSSV challenge (*p* < 0.05; Figure 1). The FM group exhibited the highest post-challenge survival rate, whereas the CAP group showed a significantly lower survival rate (*p* < 0.01). In contrast, the survival rate of the CTER group was significantly higher than that of the CAP group (*p* < 0.05) and did not differ significantly from that of the FM group (*p* > 0.05).

### 3.3. Histological Analysis of Hepatopancreas and Intestine

**Figure 2:**
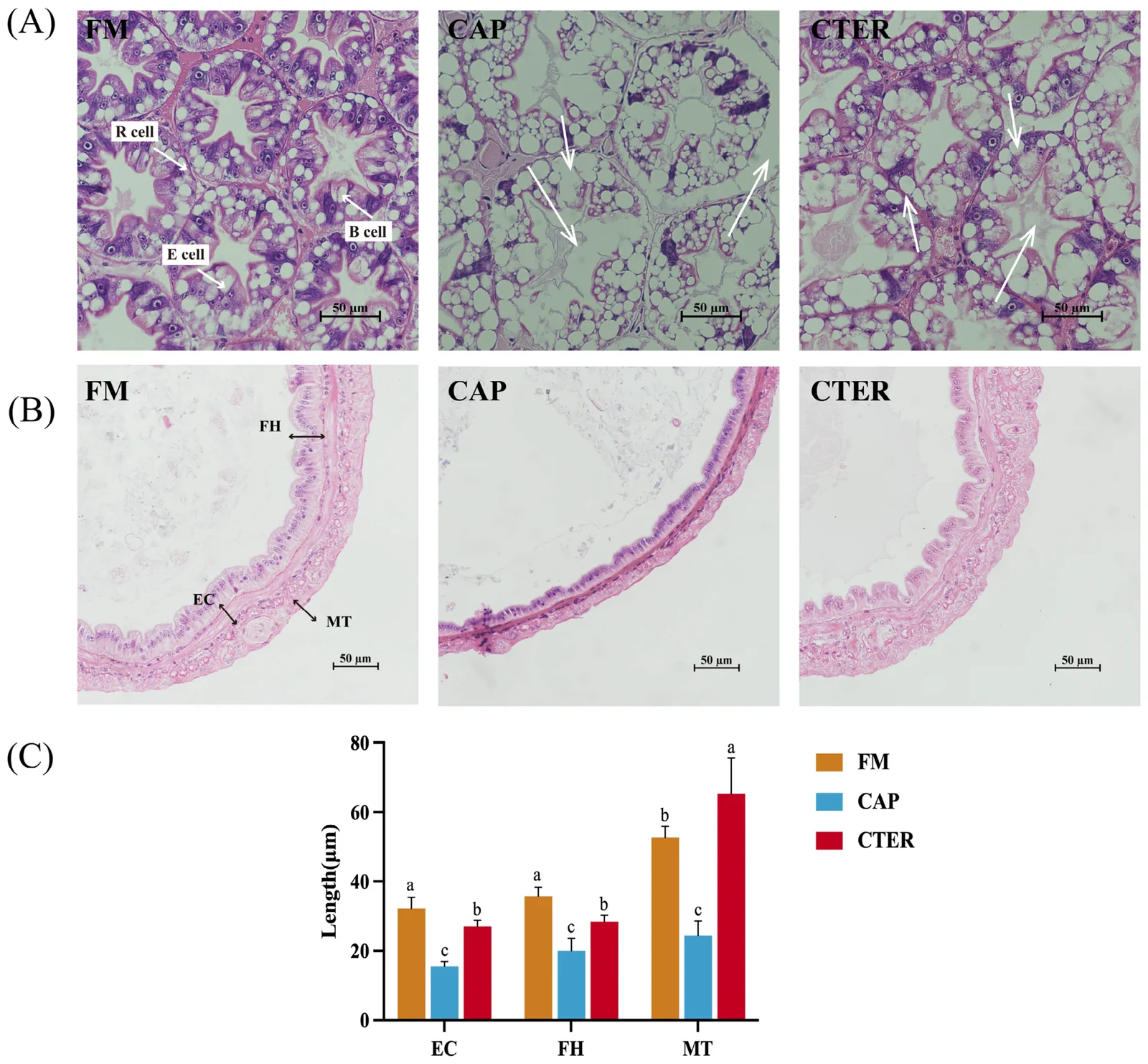
Histopathological analysis of hepatopancreas and intestinal tissues of *P. vannamei*. (**A**) (FM-CTER) represents the hepatopancreas of *P. vannamei* in the corresponding group, with a scale of 50 microns and a magnification of 40 times. B cell: blister-like cells; R cell: absorbing cells; E cell: embryonic cell; (**B**) (FM-CTER) represents the intestinal tract of *P. vannamei* in the corresponding group, with a scale of 50 microns and a magnification of 40 times; (**C**) EC: epithelial cells; FH: fold height; MT: muscle thickness. Different superscript letters denote statistically significant differences between groups (*p* < 0.05), whereas no significant difference exists between groups without letter markings. Note: Data are expressed as mean ± SD, *n* = 3. The ten measurements were regarded as technical subsamples and were averaged to obtain one representative value for each tank. The three tank-level values within each dietary treatment were used for statistical analysis.

The hepatopancreas of *P. vannamei* in the FM group was compactly arranged, with tubular structures exhibiting distinct star-shaped lumina (Figure 2A). The tubular walls comprised multiple epithelial cell types, including B, R, and E cells. In the CAP group, irregularly arranged hepatopancreatic tubules, enlarged luminal spaces, and blurred or indistinct star-shaped lumina were observed. Compared with the CAP group, the hepatopancreatic tubules in the CTER group appeared more regularly arranged, with smaller luminal spaces and more clearly defined tubular structures. In the FM group, the intestinal basement membrane and muscle layer were intact, and the epithelial cells were clearly arranged (Figure 2B). Quantitative analysis (10 measurements per section) revealed that the CAP group had flattened intestinal folds and significantly lower EC, FH, and MT than the FM group (*p* < 0.05). CUR supplementation significantly elevated these parameters in the CTER group compared with the CAP group (*p* < 0.05; Figure 2C).

### 3.4. Hepatopancreatic Non-Specific Immune and Intestinal Digestive Enzyme Activities

**Figure 3:**
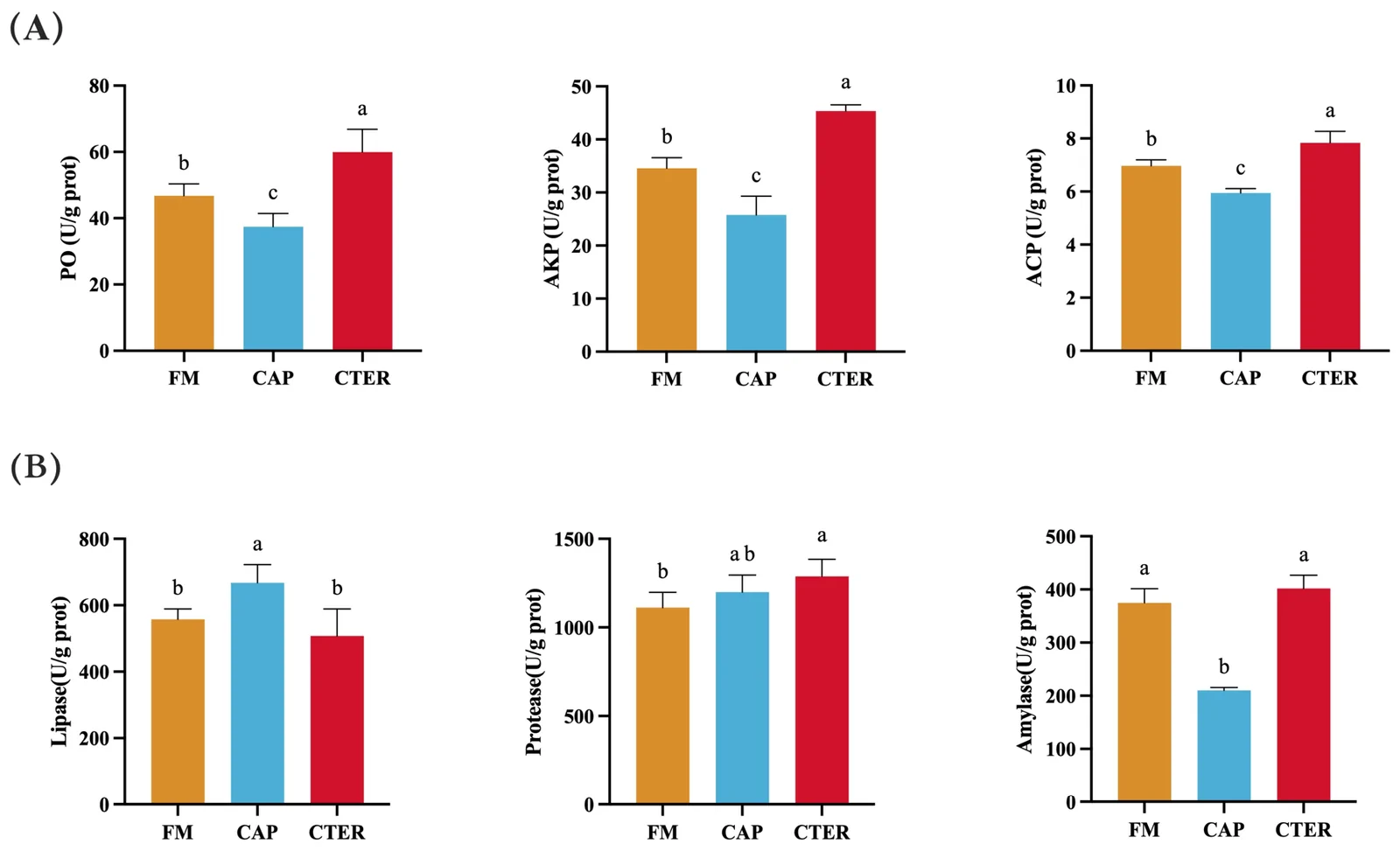
Analysis of biochemical indicators in the hepatopancreas and intestine. (**A**) Analysis of immune enzyme activity in the hepatopancreas; (**B**) Analysis of intestinal digestive enzyme activity. Different superscript letters denote statistically significant differences between groups (*p* < 0.05), whereas no significant difference exists between groups without letter markings (*p* > 0.05). Note: Data are expressed as mean ± SD, *n* = 3.

The CAP group exhibited significantly lower activities of PO, AKP, and ACP in the hepatopancreas than the FM group (*p* < 0.05; Figure 3A). In the CTER group, the activities of these three enzymes were significantly increased compared with the CAP group (*p* < 0.05) and were comparable to those in the FM group. Compared with the FM group, the CAP group showed significantly higher lipase and protease activities (*p* < 0.05; Figure 3B) but lower amylase activity (*p* < 0.05). In the CTER group, lipase activity was significantly lower than that in the CAP group (*p* < 0.05), protease activity was the highest among all groups, and amylase activity was significantly increased compared with the CAP group (*p* < 0.05), reaching a level comparable to that in the FM group.

### 3.5. Intestinal Microflora Analysis

#### 3.5.1. Intestinal Microflora Species Alpha Diversity Analysis

Good’s coverage exceeded 99% for all samples (Table 4), confirming sufficient sequencing depth. The CAP group exhibited the lowest Sobs, Chao1, and ACE indices among the three groups. The Sobs and ACE indices were significantly lower in the CAP group than in the FM and CTER groups (*p* < 0.05). The CTER group showed Sobs and ACE values similar to those of the FM group, while its Chao1 index was significantly higher (*p* < 0.05).

#### 3.5.2. Diversity Analysis of Intestinal Microbiota

**Figure 4:**
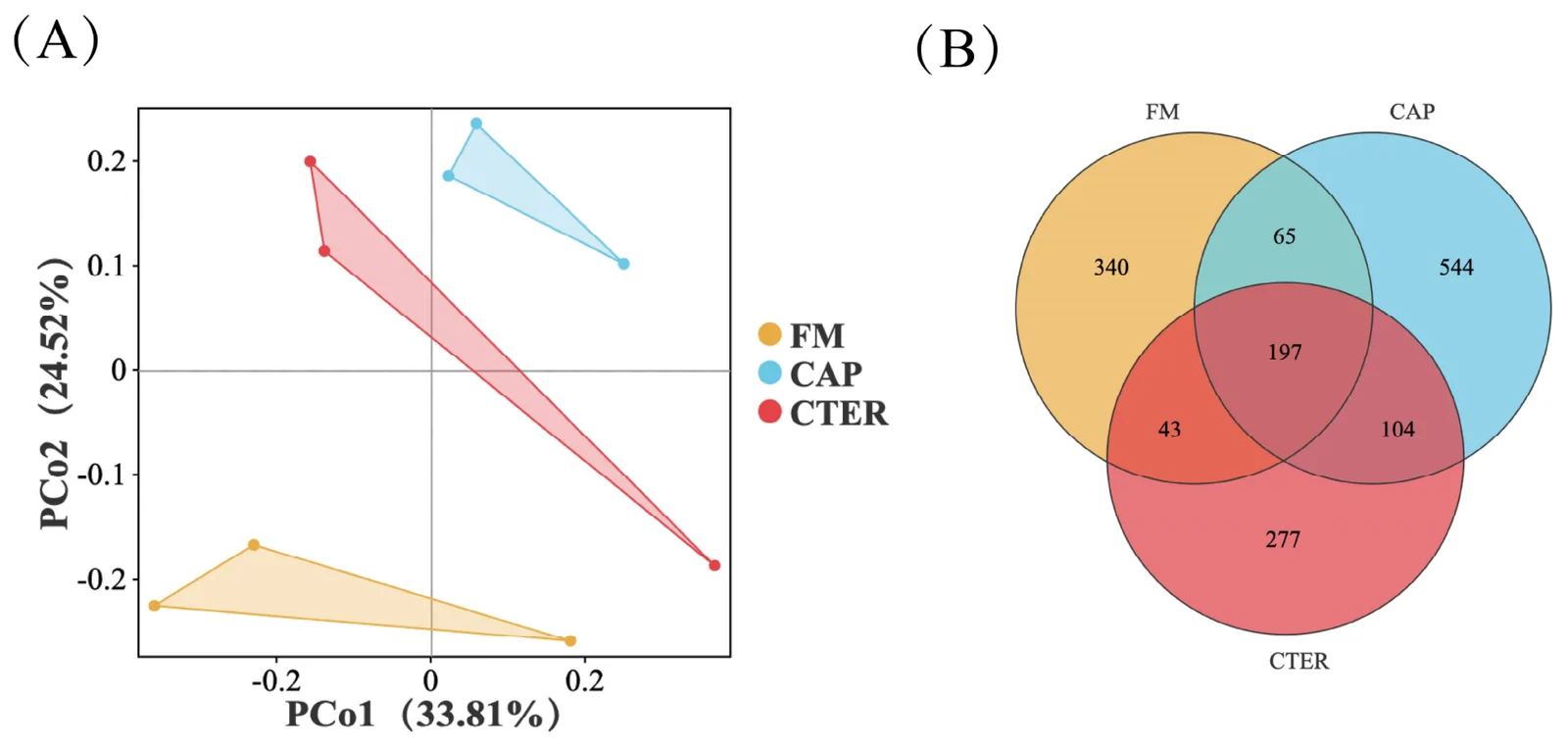
PCoA analysis (**A**) and OTU Venn diagram (**B**).

Principal coordinate analysis (PCoA) based on β-diversity was performed to assess differences in the intestinal microbiota among groups. The intestinal microbial communities of *P. vannamei* were clearly separated among the three groups, with no overlap, indicating that each dietary treatment induced a distinct shift in microbial community structure (Figure 4A). A total of 197 OTUs were shared among all samples. The FM, CAP, and CTER groups contained 340, 544, and 277 unique OTUs, respectively (Figure 4B).

#### 3.5.3. Intestinal Microbiota Composition

**Figure 5:**
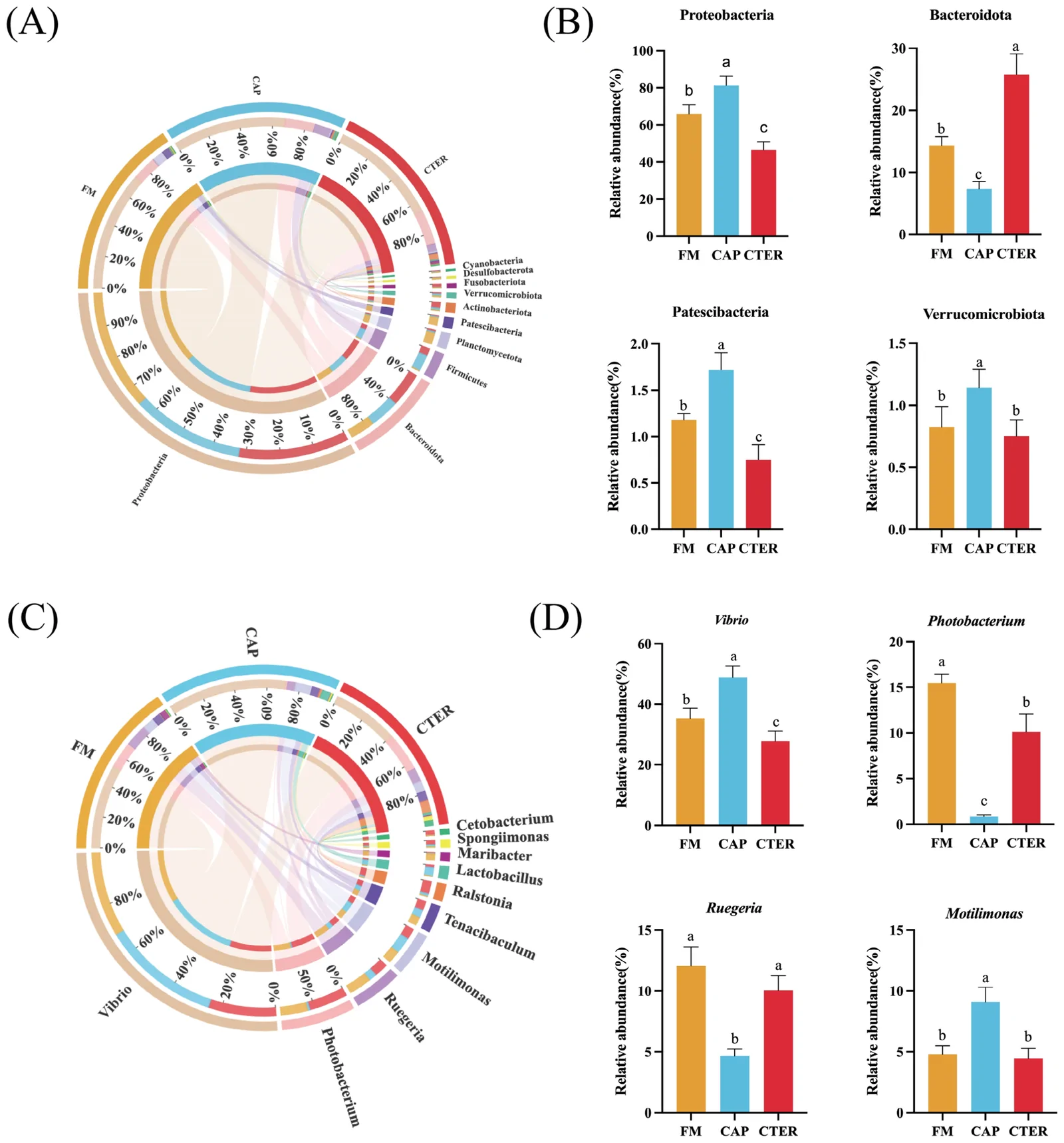
Intestinal microbiota structure and composition of *P. vannamei*. (**A**) Chord diagram of species abundance correlation at the phylum level. The first ring uses different colors to distinguish different species, while the second ring represents the proportion of each species in different groups; (**B**) Represents significantly altered and enriched microbial taxa at the phylum level; (**C**) Chord diagram of species abundance correlation at the genus level. The first ring uses different colors to distinguish different species, while the second ring represents the proportion of each species in different groups; (**D**) Represents significantly altered and enriched microbial taxa at the genus level. Different superscript letters denote statistically significant differences between groups (*p* < 0.05), whereas no significant difference exists between groups without letter markings (*p* > 0.05). Note: Data are expressed as mean ± SD, *n* = 3.

At the phylum level, the dominant bacterial phyla included Bacteroidota, Firmicutes, Planctomycetota, Actinobacteriota, Verrucomicrobiota, and Fusobacteriota (Figure 5A). The phylum-level composition of the intestinal microbiota differed among the three groups. The relative abundances of major bacterial phyla differed significantly among the three groups (Figure 5B). Compared with the FM group, the CAP group showed a significantly higher abundance of Proteobacteria but a significantly lower abundance of Bacteroidota (*p* < 0.05). In the CTER group, Proteobacteria abundance was significantly reduced and Bacteroidota abundance was significantly increased relative to the CAP group (*p* < 0.05). The CAP group also showed the highest abundances of Patescibacteria and Verrucomicrobiota, while Patescibacteria abundance was significantly reduced in the CTER group compared with the CAP group (*p* < 0.05).

At the genus level, *Vibrio* was the most abundant genus, followed by *Photobacterium*, *Ruegeria*, and *Motilimonas* (Figure 5C). The relative abundances of major bacterial genera differed significantly among the three groups (Figure 5D). *Vibrio* and *Motilimonas* showed similar patterns among groups, with the highest abundances in the CAP group and lower abundances in the CTER group. Their abundances in both the CAP and CTER groups differed significantly from those in the FM group (*p* < 0.05). *Photobacterium* abundance was significantly lower in the CAP group than in the FM group, and although it increased significantly in the CTER group compared with the CAP group, it remained significantly lower than that in the FM group (*p* < 0.05). *Ruegeria* abundance was significantly lower in the CAP group than in the FM and CTER groups (*p* < 0.05), while no significant difference was observed between the FM and CTER groups (*p* > 0.05).

#### 3.5.4. Function Prediction

**Figure 6:**
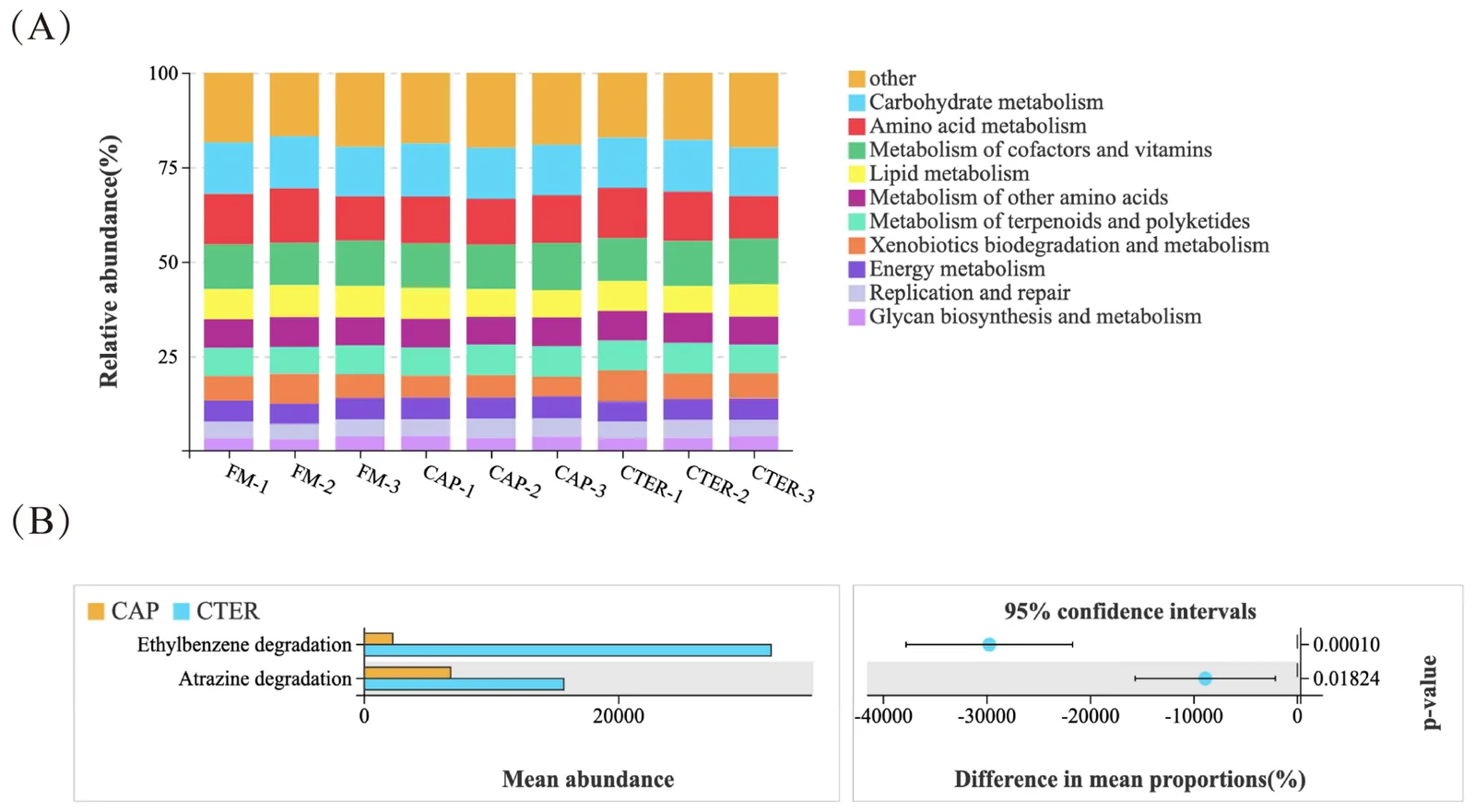
Functional predicted by PICRUSt2. (**A**) Predicted functional profiles of the intestinal microbiota based on PICRUSt2 analysis; (**B**) Differential analysis of the predicted KEGG Level 3 pathway abundances among the three groups.

The functional potential of the intestinal microbiota predicted by PICRUSt2 is shown in Figure 6A. The major predicted functional categories included carbohydrate metabolism, amino acid metabolism, metabolism of cofactors and vitamins, lipid metabolism, metabolism of terpenoids and polyketides, metabolism of other amino acids, xenobiotic biodegradation and metabolism, energy metabolism, and replication and repair. Differential analysis at KEGG Level 3 identified differences in predicted pathway abundance among the three groups (Figure 6B). Compared with the CAP group, the predicted functional abundances of KEGG pathways annotated as “ethylbenzene degradation” and “atrazine degradation” were significantly increased in the CTER group (*p* < 0.05). No significant differences were observed between the FM and CAP groups or between the FM and CTER groups (*p* > 0.05).

### 3.6. Transcriptome Analysis

#### 3.6.1. Sequencing Data Evaluation Statistics

As presented in Table 5, the proportion of high-quality reads after filtering exceeded 99%, ensuring accurate subsequent analyses. The Q20 and Q30 values were above 98% and 96%, respectively, indicating high sequencing data quality. Unique mapping rates were high across all samples, and the total mapping ratio ranged from 87% to 88%, demonstrating successful alignment of most reads to the reference genome.

#### 3.6.2. Identification of Differentially Expressed Genes

**Figure 7:**
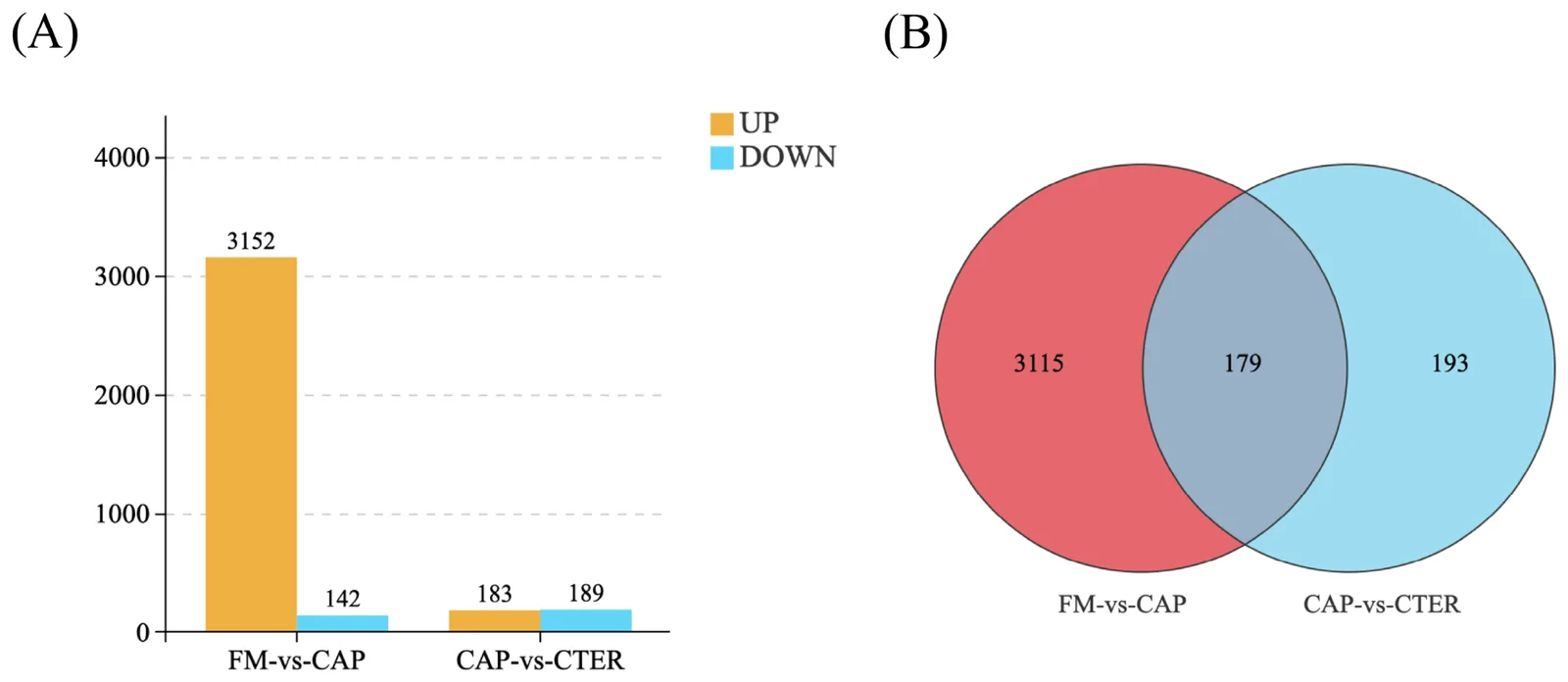
DEGs statistics and Venn diagram. (**A**) Bar chart of DEGs identified in the FM-vs.-CAP and CAP-vs.-CTER comparisons. (**B**) Venn diagram of DEGs in the FM-vs.-CAP and CAP-vs.-CTER comparisons. Overlapping regions indicated DEGs shared between groups, and nonoverlapping regions indicated DEGs specific to the group.

A total of 3294 DEGs were identified in the FM-vs.-CAP comparison, including 3152 upregulated and 142 downregulated genes, whereas 372 DEGs were identified in the CAP-vs.-CTER comparison, including 183 upregulated and 189 downregulated genes (Figure 7A). The FM-vs.-CAP comparison contained 3115 unique DEGs, and the CAP-vs.-CTER comparison contained 193 unique DEGs, with 179 DEGs shared between the two comparisons (Figure 7B).

#### 3.6.3. GO Enrichment Analysis of the DEGs

**Figure 8:**
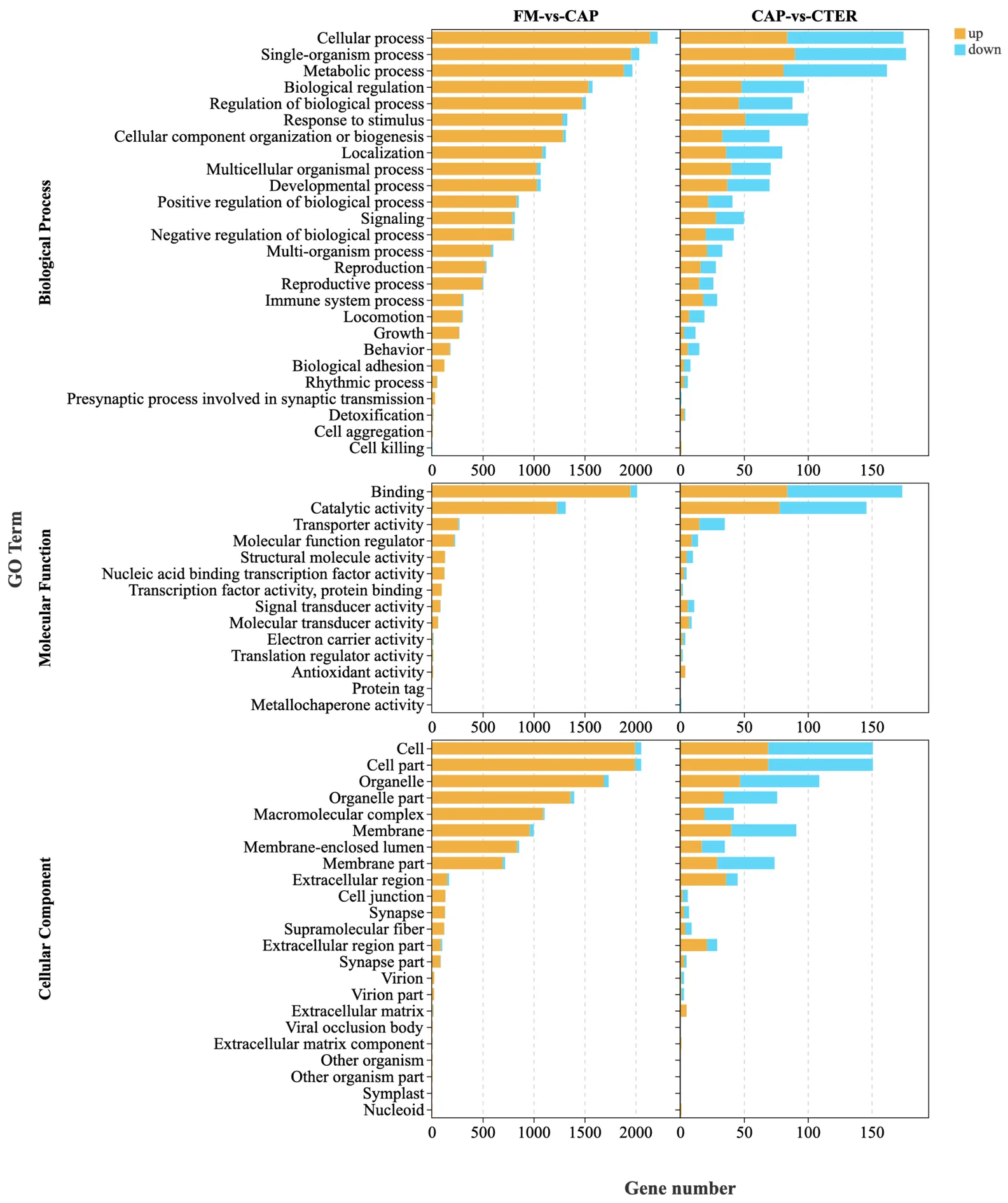
GO enrichment analysis of DEGs. Three main GO categories: cellular component, molecular function, and biological process. *X*-axis indicated the number of DEGs, *Y*-axis indicated GO categories and the subcategories.

Figure 8 displays that the distribution of enriched GO terms was similar between the two comparisons and could be classified into three major categories: Biological Processes (BP), Molecular Functions (MF), and Cellular Components (CC). In the BP category, 27 subclasses were identified, with the most enriched terms including single-organism process, cellular process, metabolic process, and biological regulation. In the MF category, 14 subclasses were identified, and the most enriched terms were binding and catalytic activity. In the CC category, 23 subclasses were identified, mainly including cell, cell part, organelle, and protein-containing complex.

#### 3.6.4. KEGG Enrichment Analysis of the DEGs

**Figure 9:**
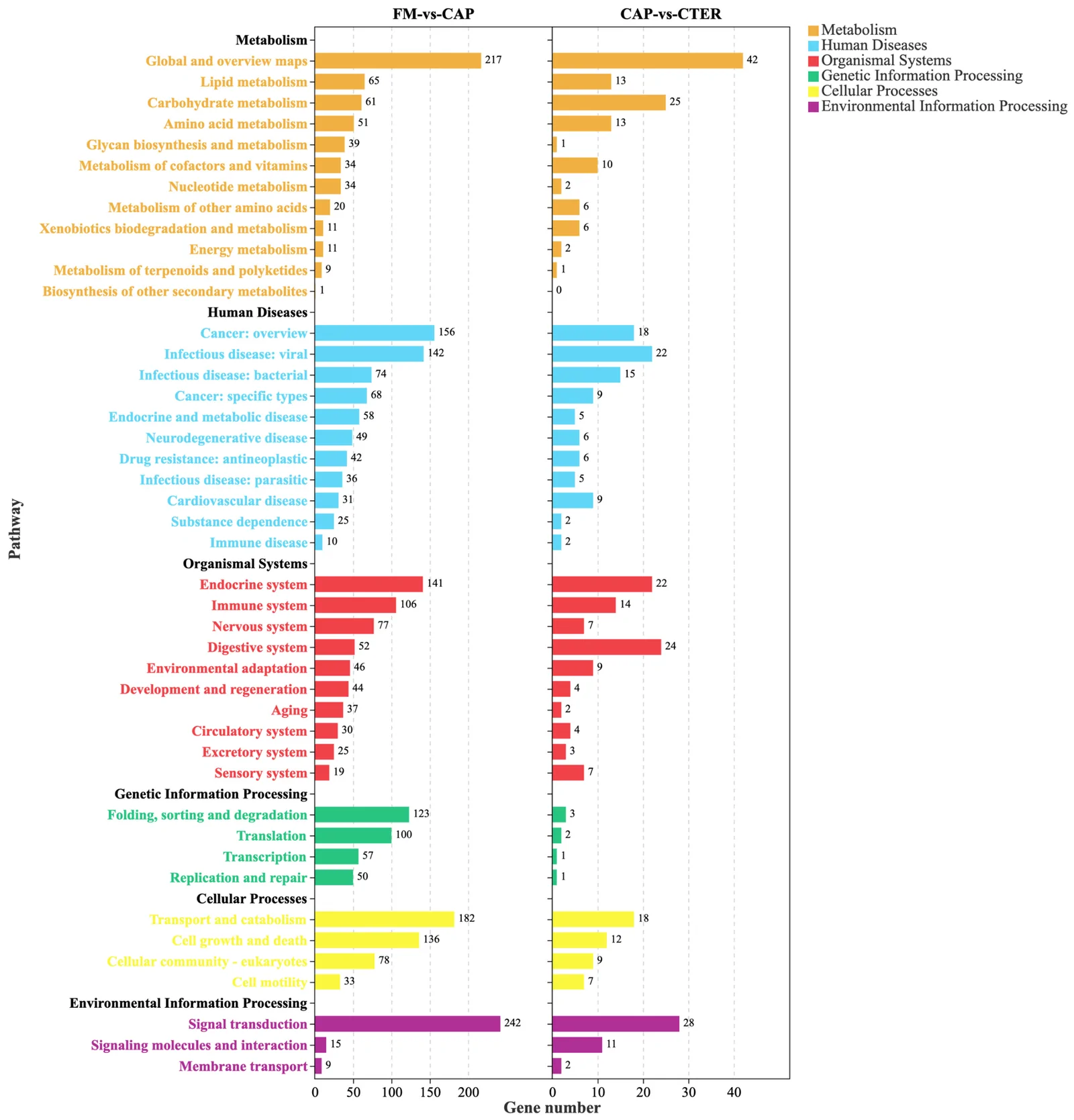
KEGG enrichment analysis of DEGs. Using the KEGG pathway database, which is a metabolic pathway database, biological metabolic pathways are classified into six categories: Metabolism, Human Diseases, Organismal Systems, Genetic Information Processing, Cellular Processes, and Environmental Information Processing. The predicted functions in the major classes were ranked in order of relative abundance.

KEGG enrichment analysis was performed to identify significantly enriched pathways among the DEGs identified in the FM-vs.-CAP and CAP-vs.-CTER comparisons (Figure 9). The enriched pathways were classified into six major categories: metabolism, human diseases, organismal systems, genetic information processing, cellular processes, and environmental information processing. In the metabolism class, DEGs from both comparisons were mainly enriched in lipid and carbohydrate metabolism, as well as cofactor and vitamin metabolism. In the KEGG “Human Diseases” category, DEGs from the FM-vs.-CAP and CAP-vs.-CTER comparisons were mapped to several database annotation categories related to cancer and infectious diseases, respectively. These KEGG category labels reflect shared genes and signaling components. In organismal systems, the most enriched pathways were the endocrine, immune, and digestive systems. In the other three categories, the most enriched pathways were folding/sorting/degradation, transport and catabolism, and signal transduction.

**Figure 10:**
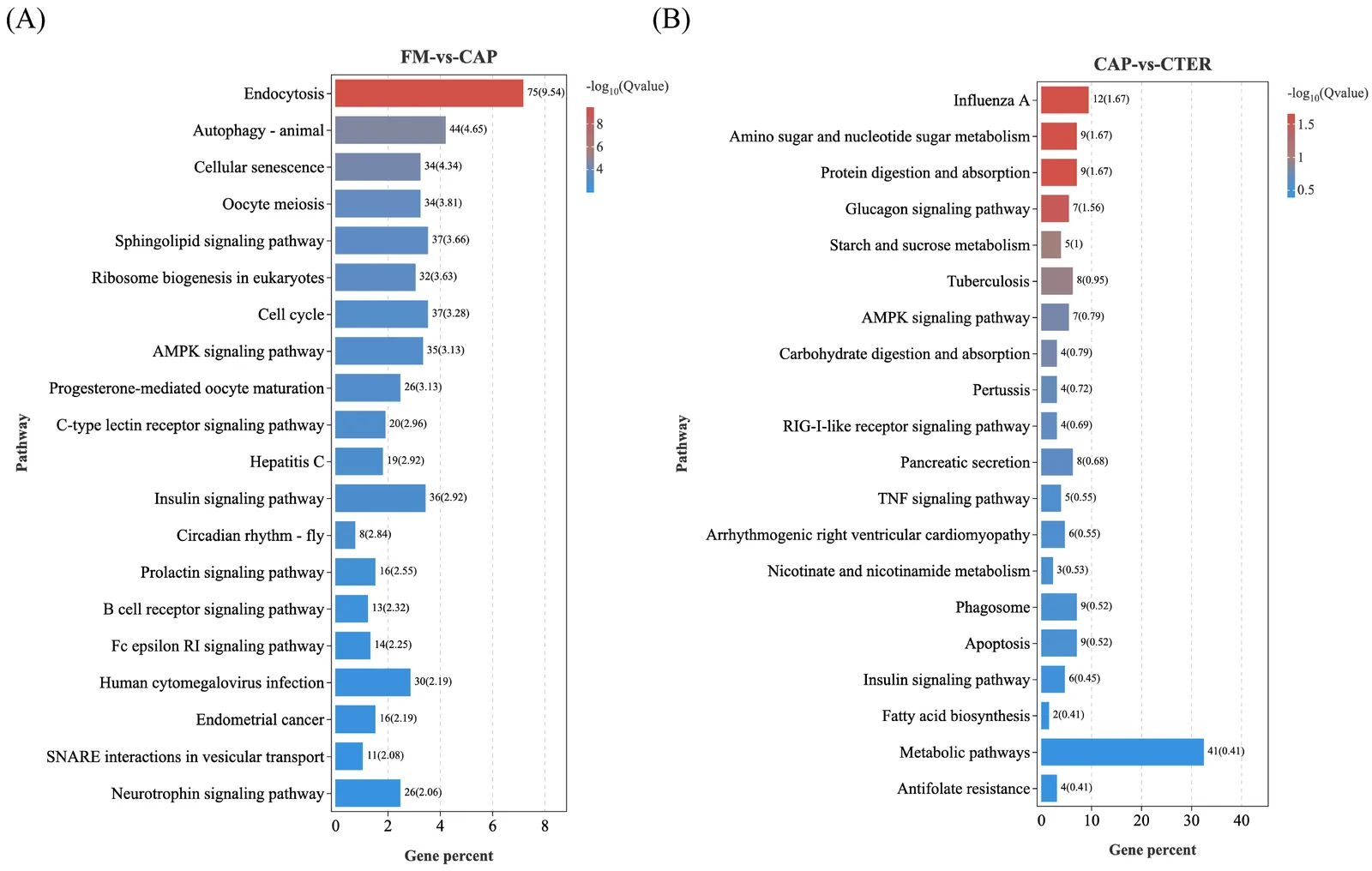
Top 20 pathways in KEGG enrichment analysis. (**A**) Top 20 KEGG enriched pathways of DEGs in the FM-vs.-CAP comparison; (**B**) Top 20 KEGG enriched pathways of DEGs in the CAP-vs.-CTER comparison.

Among the top 20 enriched KEGG pathways in the FM-vs.-CAP comparison, differentially expressed genes (DEGs) were mapped to pathways including endocytosis, autophagy, cellular senescence, and oocyte meiosis. DEGs were also mapped to metabolism-related pathways, including the AMPK and insulin signaling pathways (Figure 10A). In the CAP-vs.-CTER comparison, DEGs were mapped to KEGG annotation categories labeled “Influenza A” and “Tuberculosis”, as well as to the TNF signaling pathway. In addition, DEGs were mapped to metabolism-related pathways, including amino sugar and nucleotide sugar metabolism, carbohydrate digestion and absorption, and the insulin and AMPK signaling pathways (Figure 10B). Overall, DEGs were mapped to the AMPK and insulin signaling pathways in both the FM-vs.-CAP and CAP-vs.-CTER comparisons (Table 6).

#### 3.6.5. Validation of the RNA-seq Results by qPCR

**Figure 11:**
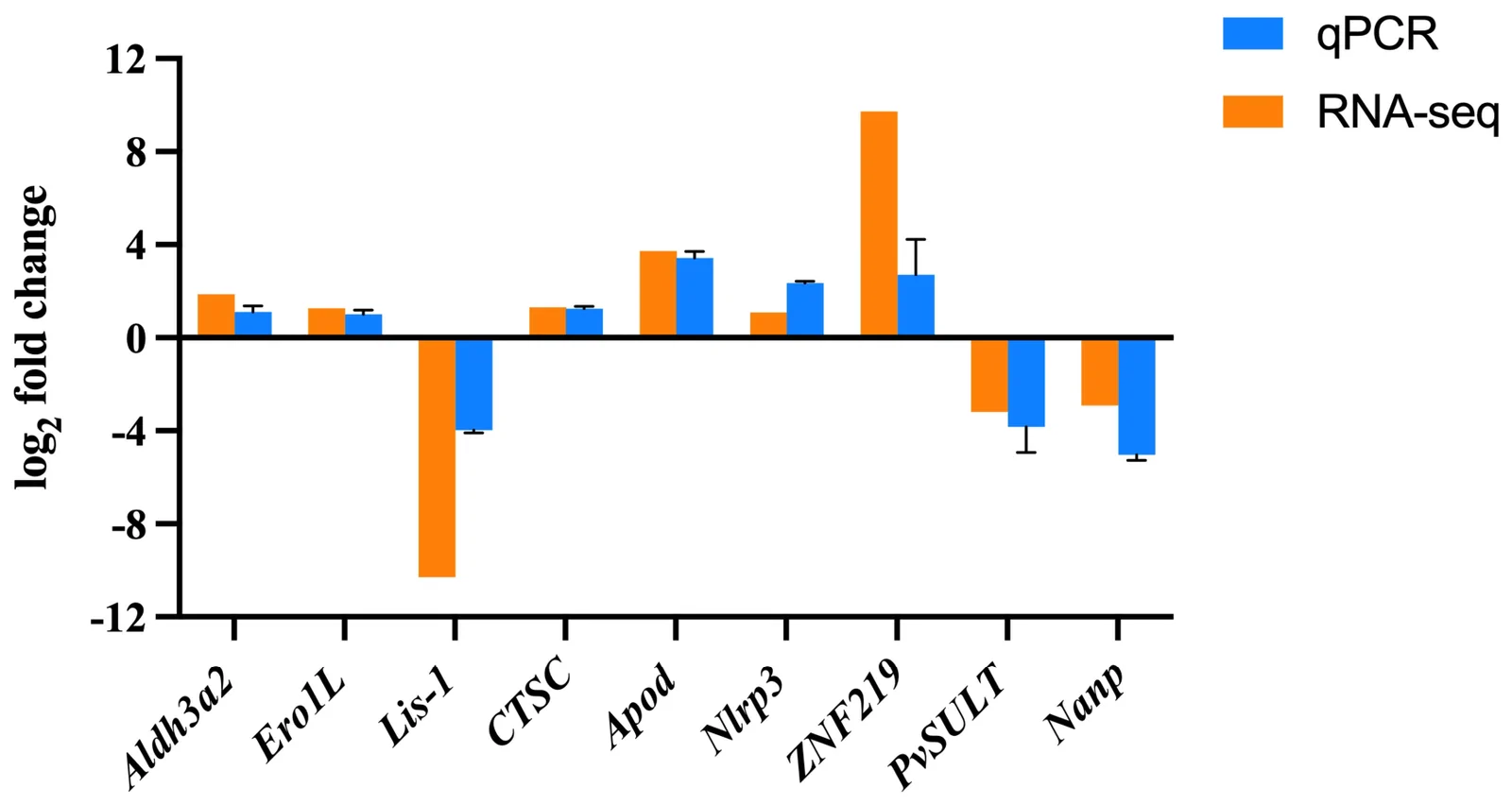
qPCR validation of DEGs. The detection of gene expression was performed in triplicate for each sample. Expression values were normalized to the geometric mean of *EF-1α* using the Livak (2^−ΔΔCt^) method, and the data are presented as the means ± SD of triplicate assays.

Nine DEGs, including six up-regulated and three down-regulated genes, were selected to validate the RNA-seq results. The qPCR results showed expression patterns consistent with the RNA-seq data, supporting the reliability of the transcriptome sequencing results (Figure 11). Pearson correlation analysis further revealed a significant positive correlation between the log2 fold-change values obtained by RNA-seq and qPCR (r = 0.807, R^2^ = 0.651, *p* = 0.0086), further supporting the reliability of the transcriptome sequencing results.

## 4. Discussion

The present study investigated whether dietary CUR could mitigate the adverse effects of high-level fishmeal replacement with CAP in *P. vannamei*. Under the present dietary conditions, high CAP inclusion adversely affected growth and several health-related parameters, whereas CUR supplementation was associated with improvements in growth performance, tissue condition, immune responses, intestinal microbiota, and transcriptomic profiles. The experiment was designed as a targeted nutritional intervention based on published evidence concerning the general biological effects of curcumin in *P. vannamei*, together with preliminary dose–response observations from the author’s graduate research. Accordingly, the primary objective was to evaluate the effects of CUR under high-CAP dietary conditions rather than to re-examine its general effects in a conventional fishmeal-based diet. Nevertheless, because an FM+CUR group was not included, the present design cannot formally distinguish general curcumin-mediated benefits from CAP-specific alleviating effects or evaluate the CAP × curcumin interaction. Therefore, the observed benefits should be interpreted specifically within the context of high-CAP dietary conditions.

The rising scarcity and price volatility of FM have driven the search for alternative proteins like CAP in aquafeed production. Nevertheless, high inclusion levels of CAP frequently compromise the growth performance of aquatic animals. Specifically, Yao et al. reported that replacement of fishmeal with CAP at levels of 45%, 70%, and 100% significantly reduced the growth performance of *P. vannamei*, while the FCR was significantly higher than that of the control group [20]. The present findings indicate that replacing 50% of fishmeal with CAP impaired the growth performance of *P. vannamei*, particularly in terms of WGR and SGR. Previous studies have suggested that growth suppression associated with high-level CAP substitution may be related to reduced feed palatability, altered nutrient availability, and changes in amino acid balance, which may subsequently affect feed intake, intestinal development, and nutrient utilization [21,43]. However, because dietary amino acid profiles, feed intake, and nutrient digestibility were not directly determined in the present study, these mechanisms remain speculative. In our study, the reduced growth performance in the CAP group was accompanied by impaired intestinal morphology, altered digestive enzyme activities, and disturbances in metabolism-related pathways, which may have contributed to reduced nutrient utilization. Notably, CUR supplementation restored the growth performance of *P. vannamei* fed the CAP-based diet to levels comparable to those of the FM group. Comparable protective effects have been reported for other functional additives in low-fishmeal diets: yeast-derived β-glucan and mannan oligosaccharides enhanced biomass, nonspecific immunity, and resistance to *Vibrio parahaemolyticus*, whereas co-cultured *Lactobacillus acidophilus* and *Bacillus subtilis* improved growth, hepatopancreatic integrity, immune status, and stress resistance in *P. vannamei* [44,45]. Similarly, supplementation with quercetin, another dietary polyphenol, improved growth, antioxidant and immune capacity, intestinal morphology, and microbial homeostasis in juvenile Chinese mitten crabs fed a low-fishmeal diet [46]. These findings indicate that CUR exhibits a broad protective profile comparable to those of prebiotics, probiotics, and other polyphenols.

In both *P. vannamei* and *P. clarkii*, substituting 25–50% of dietary FM with CAP stimulated immune activity and improved defense against external challenges [21,47]. However, substitution exceeding 50% resulted in immunosuppression. A possible explanation is that CAP supplies not only protein but also vitamins, minerals, and cell wall constituents, which may serve as immunostimulatory agents that trigger immune responses. Fishmeal contains bioactive compounds—including small peptides and taurine—that participate in immune regulation, but these are absent in CAP. Consequently, replacing too much FM with CAP directly compromises the host’s immune capacity [48]. In this study, *P. vannamei* in the CAP group showed lower survival following WSSV exposure than those in the FM group, whereas CUR supplementation was associated with higher post-challenge survival than the CAP diet alone. The activities of hepatopancreatic nonspecific immune enzymes also increased in the CUR-supplemented group. However, the challenge inoculum consisted of a crude WSSV-infected tissue extract, and WSSV infection or viral load was not subsequently confirmed by qPCR. Consequently, the survival results should be regarded as supportive evidence of differences in post-challenge survival rather than definitive evidence of treatment effects on WSSV resistance or viral replication.

Since crustaceans lack adaptive immunity, their defense mechanisms rely primarily on innate immunity, in which immune enzymes such as PO, AKP, and ACP are commonly used as nonspecific immune indicators [49,50]. It has been well documented that high-level substitution of fishmeal with alternative proteins frequently impairs immune homeostasis and reduces the host’s ability to resist pathogenic infection [51,52,53,54]. Consistent with these observations, the present study found that the activities of PO, AKP, and ACP in the CAP group were significantly lower than those in the FM group, indicating reduced activities of the measured nonspecific immune enzymes under the high-CAP dietary condition. Interestingly, after supplementing with CUR, the immune indicators of shrimp were significantly improved. These results suggest that CUR supplementation was associated with improvements in the measured nonspecific immune enzyme activities of *P. vannamei* under the high-CAP dietary condition. However, these enzymes are not specific markers of antiviral immunity and cannot demonstrate enhanced disease resistance, suppression of WSSV replication, or activation of defined antiviral pathways.

The crustacean hepatopancreas is a multifunctional organ that performs digestive, nutrient-storage, and detoxification functions analogous to those of the vertebrate liver and pancreas [55,56]. Because it is highly responsive to dietary alterations, its structural integrity and cellular composition can provide information on the physiological status of shrimp [57,58]. Similar to the hepatopancreas, the intestinal morphology of shrimp can also reflect its nutritional status and overall health. Similarly, intestinal morphology is commonly used to evaluate intestinal condition and nutritional responses in shrimp [59]. In the present study, shrimp in the CAP group exhibited adverse histological changes in the hepatopancreas and intestine, consistent with previous reports on high-level fishmeal replacement [60,61]. Compared with the CAP group, CUR supplementation was associated with improvements in hepatopancreatic and intestinal morphology. CUR supplementation was also associated with changes in the activities of lipase, protease, and amylase in the intestine. However, these parallel responses do not demonstrate that CUR directly stimulated digestive-enzyme secretion, repaired tissue damage, or enhanced immune capacity. This explanation is consistent with previous research [62]. Overall, the results indicate that CUR supplementation was associated with improved tissue morphology and digestive enzyme activities under the high-CAP dietary condition.

Recent studies have demonstrated that the gut microbiota not only contributes to improved nutrient utilization and host growth and development [63], but also enhances disease resistance by modulating the immune system and improving defenses against pathogenic microorganisms [64]. In this study, high-level fishmeal replacement was associated with marked changes in the intestinal microbiota of *P. vannamei*. Compared with the FM group, the CAP group exhibited higher relative abundances of Proteobacteria and *Vibrio*, together with lower relative abundances of several dominant bacterial taxa, including Bacteroidota, *Ruegeria*, and *Motilimonas*. These compositional shifts may reflect a disruption of intestinal microbial homeostasis in shrimp [65,66]. One possible explanation for the increased abundances of Proteobacteria and *Vibrio* is that high-level CAP substitution impaired intestinal structural integrity and altered the nutritional environment, creating conditions favorable for opportunistic bacteria [67]. This is supported by the observed intestinal fold flattening, altered digestive enzyme activities, and reduced microbial richness in the CAP group. Additionally, the CAP-induced reduction in *Ruegeria*, a genus with reported antagonistic activity against *Vibrio* [68], may have weakened competitive suppression and facilitated Vibrio expansion. Overall, the microbial shifts in the CAP group may have been associated with altered intestinal conditions, dietary substrates, and bacterial interactions. Interestingly, the abundance of pathogenic *Photobacterium* decreased significantly in the CAP group, while that of Verrucomicrobiota increased markedly. Because 16S rRNA profiling cannot determine whether individual strains are pathogenic or beneficial, these results should be interpreted only as differences in taxonomic composition. This observation may reflect differential ecological functions and nutritional requirements among bacterial taxa, leading to divergent responses to fishmeal replacement stress. The underlying mechanisms warrant further investigation. Following CUR supplementation, the intestinal microbiota structure of shrimp underwent a favorable transformation, returning to levels comparable to those of the FM group and exhibiting greater stability. Collectively, these results suggest that CUR supplementation was associated with changes in intestinal microbial community composition under the CAP-based dietary condition.

Transcriptomic analysis provided additional molecular context for the physiological responses of *P. vannamei* to high-level fishmeal replacement with CAP and CUR supplementation. A total of 3294 DEGs were identified in the FM-vs.-CAP comparison, whereas 372 DEGs were detected in the CAP-vs.-CTER comparison, with 179 DEGs shared between the two comparisons. The relatively large number of DEGs in the FM-vs.-CAP comparison may reflect a broad hepatopancreatic transcriptional response to the marked change in dietary protein source, whereas the smaller set of DEGs in the CAP-vs.-CTER comparison may represent more selective transcriptional adjustments following CUR supplementation. GO analysis showed that DEGs from both comparisons were mainly assigned to cellular and metabolic processes, biological regulation, binding, and catalytic activity, suggesting that CAP replacement and CUR supplementation may influence partially overlapping gene networks related to nutrient metabolism, cellular maintenance, and physiological adaptation. Consistent with these findings, Jiang et al. reported that high CAP inclusion in *P. vannamei* was accompanied by reduced growth and changes in genes associated with pancreatic secretion, protein digestion and absorption, ribosomal function, phagosomes, and lysosomes [21]. Yao et al. also found that replacing at least 45% of dietary fishmeal with CAP reduced shrimp growth and was associated with changes in amino acid and fatty acid metabolism [20]. Similar responses have been observed in other aquatic species: excessive CAP replacement reduced protein and lipid digestibility and altered postprandial serum free-amino-acid profiles in obscure pufferfish [48], while high CAP inclusion in grow-out turbot was associated with reduced feed intake, altered plasma lipid indices, and changes in hepatic genes related to lipid synthesis, transport, and oxidation [69]. These comparable findings suggest that excessive CAP inclusion may be associated with changes in nutrient digestion and metabolic utilization, although nutrient availability and metabolic flux were not directly measured in the present study. KEGG analysis identified DEGs annotated to AMPK, insulin, autophagy, and sphingolipid-related pathways among those enriched with DEGs in the FM-vs.-CAP and CAP-vs.-CTER comparisons. Previous studies in crustaceans have associated AMPK- and autophagy-related responses with nutrient sensing, glucose and lipid metabolism, cellular recycling, and adaptation to metabolic or environmental stress [70,71,72]. When considered together with the reduced growth performance, hepatopancreatic and intestinal damage, and decreased PO, AKP, and ACP activities in the CAP group, these enrichment patterns suggest associations between the observed transcriptional changes and processes related to nutrient utilization, energy metabolism, cellular maintenance, and innate immune function. However, these associations do not demonstrate functional activation or inhibition of the corresponding pathways, and further validation at the protein or functional level is required. In the CAP-vs.-CTER comparison, DEGs were enriched in pathways associated with AMPK and insulin signaling, carbohydrate digestion and absorption, amino sugar and nucleotide sugar metabolism, TNF signaling, and nicotinate and nicotinamide metabolism. These transcriptional changes were broadly consistent with the improved growth performance, alleviated tissue damage, increased immune enzyme activities, and higher survival following WSSV exposure in the CTER group. Previous studies in *P. vannamei* have similarly reported that dietary CUR preparations may improve growth, digestive function, antioxidant and immune status, intestinal condition, and resistance to pathogenic challenge [34,35,36,62]. Moreover, CUR supplementation in a high plant-protein diet improved growth, antioxidant capacity, and hepatopancreatic and intestinal condition in *Macrobrachium rosenbergii*, accompanied by changes in transcriptomic and metabolomic profiles [39]. A curcuminoid-containing botanical preparation also improved growth, immune responses, and survival following WSSV challenge in *P. vannamei* [73]. The enrichment of nicotinate and nicotinamide metabolism may additionally reflect changes in NAD^+^-related cofactor metabolism and redox-associated processes following CUR supplementation [74]. Overall, the integrated transcriptomic and physiological findings suggest that CUR supplementation was associated with coordinated transcriptional changes in genes related to nutrient utilization, energy metabolism, cellular maintenance, and innate immune responses, which may help explain the improved growth and health-related phenotypes of *P. vannamei* fed the high-CAP diet.

However, because a fishmeal control diet supplemented with curcumin was not included in the present experimental design, we cannot distinguish CAP-specific rescue effects from the general beneficial effects of curcumin. Therefore, the beneficial effects observed in this study should be interpreted specifically in the context of high-CAP dietary conditions. In addition, the nutritional interpretation of the present results is limited by the absence of detailed amino-acid profiles, taurine concentrations, digestible energy estimates, and nucleic-acid or nucleotide measurements for CAP and the complete diets. Therefore, possible differences in nutrient supply among the diets cannot be excluded. Moreover, because CUR recovery, mixing homogeneity, and storage stability were not analytically verified, the actual CUR exposure may have differed from the nominal inclusion level, particularly because partial thermal loss during feed processing may have occurred. Accordingly, the proposed nutritional explanations should be regarded as hypothetical and require further analytical verification.

## 5. Conclusions

High-level replacement of fishmeal with CAP adversely affected the growth performance, intestinal and hepatopancreatic morphology, nonspecific immune enzyme activities, and intestinal microbial community composition of *P. vannamei*. Compared with the CAP group, supplementation with CUR (60 mg/kg) was associated with improved growth performance, intestinal and hepatopancreatic morphology, digestive enzyme activities, nonspecific immune responses, microbial richness, and survival following WSSV exposure. CUR supplementation was also associated with changes in the relative abundance of several dominant bacterial taxa, including a lower relative abundance of *Vibrio*. Transcriptomic analysis showed that CUR supplementation was associated with differential expression of genes enriched in pathways related to metabolism and immunity. Overall, the findings support the potential application of CUR as a functional additive in CAP-based, low-fishmeal diets for *P. vannamei*. Nevertheless, the absence of an FM+CUR group limits our ability to distinguish the specific protective effects of curcumin against CAP-induced nutritional stress from its potential general beneficial effects.

## Figures and Tables

**Table 1:** Composition and nutrient levels of the experimental diets (dry weight, %).

Ingredients	Groups		
	FM	CAP	CTER
Red fish meal	25.00	12.50	12.50
*Clostridium autoethanogenum* Protein	0.00	10.98	10.98
Soybean meal	18.00	18.00	18.00
Shrimp shell meal	4.00	4.00	4.00
Peanut meal	7.00	7.00	7.00
Corn protein meal	8.00	8.00	8.00
Flour	25.00	25.00	25.00
Fish oil	2.00	2.92	2.92
Corn oil	2.00	2.00	2.00
Soy phospholipids	0.50	0.50	0.50
Choline chloride	0.50	0.50	0.50
Ethoxyquinoline	0.03	0.03	0.03
Dimethyl-β-propiothetin	0.10	0.10	0.10
CaHPO_4_	1.50	1.50	1.50
Microcrystalline cellulose	5.62	6.22	6.214
Multi-vitamin ^a^	0.20	0.20	0.20
Mineral premix ^b^	0.50	0.50	0.50
Vitamin C	0.05	0.05	0.05
Curcumin	0.00	0.00	0.006
Total	100.00	100.00	100.00
Nutrient compositions of experimental diets (in dry matter, %)			
Crude protein	44.62	43.93	44.58
Crude lipid	6.91	6.33	6.49
Crude ash	9.60	8.88	8.83
Moisture	8.78	10.89	9.71

Note: ^a^ Multidimensional premix is provided per kilogram of feed: VC 150 mg, VE 40 mg, VA 5000 IU, VD3 1000 IU, thiamine 5 mg, riboflavin 10 mg, menadione 10 mg, pyridoxine 10 mg, biotin 0.1 mg, cyanocobalamin 0.02 mg, calcium pantothenate 20 mg, folic acid 1 mg, nicotinic acid 40 mg. ^b^ Mineral premixes are provided per kilogram of feed: FeSO_4_·H_2_O 303 mg, KIO_3_ 1.3 mg, Cu_2_(OH)_3_Cl 5 mg, ZnSO_4_·H_2_O 138 mg, MnSO_4_·H_2_O 36 mg, Na_2_SeO_3_ 0.6 mg, CoCl_2_·6H_2_O 0.8 mg.

**Table 2:** qPCR primer sequences.

Genes	Forward (5′-3′)	Reverse (5′-3′)	Gene Bank No.
*EF1-α*	TGCACCACGAAGCCCTTAC	CAGGGTGGTTGAGGACGATC	XM_027373349.1
*Aldh3a2*	TTGGTGGAGAAGTGAATAA	TGAAGTTGATAGCCTCGTA	XM_027365552.1
*Ero1L*	TTCCAGAGGGCATAAAGGG	TCACAGTGTCCAGGCACCA	XM_027368643.1
*Lis-1*	GCCTCTTCACTCTGGTTGG	TTGGCATCGTTTGTTCTTTA	XM_027355967.1
*CTSC*	CACCAAGACCTTCACCCTC	TGGTTCTCATTCTGGTGGTT	XM_027380508.1
*Apod*	CCCAACAACGAATACATCA	CAGAAGGGAAATCAACGAG	XM_027377490.1
*Nlrp3*	AGAACGAGCATTTCCACAG	ATGATCCATTGAACGCAGT	XM_027383679.1
*ZNF219*	CACTGTAACTATGCTGCCTCT	GAGAACTCGCCTTGGTAAA	XM_027381214.1
*PvSULT*	CCCTTGTTGCTCTTGAATC	GAGAACTCGCCTTGGTAAA	XM_027375805.1
*Nanp*	CAGGTCCGTCCCTCTGAAT	TTGAAGTTTACCATTTGCTGCATAA	XM_027364599.1

Note: *EF-1α* was used as the reference gene. *EF-1α*, elongation factor 1-alpha; *Aldh3a2*, aldehyde dehydrogenase 3 family member A2; *Ero1L*, endoplasmic reticulum oxidoreductase 1 alpha; *Lis-1*, Lissencephaly-1; *CTSC*, cathepsin C; *Apod*, apolipoprotein D; *Nlrp3*, NLR family pyrin domain containing 3; *ZNF219*, zinc finger protein 219; *PvSULT*, *Penaeus vannamei* sulfotransferase 1C4-like; *Nanp*, N-acylneuraminate-9-phosphatase.

**Table 3:** Growth performance of *P.vannamei*.

Groups	FBW/g	SR/%	WGR/%	SGR/(%/d)	FCR
FM	14.44 ± 0.15 ^a^	90.00 ± 2.50	5347.34 ± 55.88 ^a^	7.14 ± 0.02 ^a^	1.51 ± 0.03
CAP	12.26 ± 0.07 ^b^	87.50 ± 2.50	4526.27 ± 28.28 ^b^	6.85 ± 0.01 ^b^	1.62 ± 0.08
CTER	14.24 ± 0.09 ^a^	91.67 ± 1.44	5274.27 ± 34.00 ^a^	7.11 ± 0.01 ^a^	1.58 ± 0.06

Note: Within the same column, data marked with no superscript letters, or the same superscript lowercase letter, indicate no significant differences between groups (*p* > 0.05), while distinct lowercase letters denote statistically significant differences (*p* < 0.05). The same convention applies to subsequent tables.

**Table 4:** The α-diversity of intestinal microbiota in *P. vannamei*.

Groups	Items					
	Good’s Coverage	Sobs	Shannon	Simpson	Chao1	ACE
FM	0.99 ± 0.00	529.67 ± 10.02 ^a^	4.35 ± 0.41	0.88 ± 0.07	555.65 ± 12.41 ^b^	582.62 ± 12.18 ^a^
CAP	0.99 ± 0.00	473.33 ± 16.50 ^b^	4.11 ± 0.44	0.86 ± 0.05	539.36 ± 8.19 ^b^	526.62 ± 31.76 ^b^
CTER	0.99 ± 0.00	507.00 ± 7.00 ^a^	4.76 ± 0.41	0.91 ± 0.04	581.58 ± 16.27 ^a^	601.13 ± 27.56 ^a^

Notes: Good’s coverage: Sampling completeness; Sobs: Observed OTU/ASV richness; Shannon: Species diversity (evenness + richness); Simpson: Species dominance; Chao1: Chao1 estimated richness; ACE: ACE estimated richness. Within the same column, data marked with no superscript letters, or the same superscript lowercase letter indicate no significant differences between groups (*p* > 0.05), while distinct lowercase letters denote statistically significant differences (*p* < 0.05). Data are expressed as mean ± SD, *n* = 3.

**Table 5:** Statistics of transcriptome sequencing in *P.vannamei*.

Sample	Raw Read	Clean Read (%)	Clean Data (bp)	Q20 (%)	Q30 (%)	GC (%)	Unique Map	Mult Map	Total Map
FM-1	44,572,154	44,389,234 (99.59%)	6,597,954,947	98.88%	96.80%	48.90%	29,603,884 (69.05%)	8,017,237 (18.70%)	37,621,121 (87.74%)
FM-2	41,930,144	41,787,428 (99.66%)	6,185,372,204	98.92%	96.86%	49.35%	27,510,950 (67.66%)	8,594,355 (21.14%)	36,105,305 (88.79%)
FM-3	46,871,442	46,663,746 (99.56%)	6,927,656,553	98.83%	96.62%	50.65%	31,321,057 (68.35%)	9,031,230 (19.71%)	40,352,287 (88.06%)
CAP-1	53,063,186	52,798,062 (99.50%)	7,819,616,434	98.93%	96.90%	48.39%	35,959,082 (70.63%)	8,821,440 (17.33%)	44,780,522 (87.96%)
CAP-2	44,199,534	44,003,908 (99.56%)	6,525,442,904	98.85%	96.73%	49.02%	30,056,036 (70.57%)	7,158,867 (16.81%)	37,214,903 (87.38%)
CAP-3	45,922,732	45,760,562 (99.65%)	6,787,282,259	98.89%	96.79%	47.88%	31,294,357 (71.15%)	7,450,619 (16.94%)	38,744,976 (88.09%)
CTER-1	58,326,476	58,084,996 (99.59%)	8,588,301,307	98.92%	96.92%	48.70%	39,560,940 (70.66%)	9,783,970 (17.48%)	49,344,910 (88.14%)
CTER-2	50,968,282	50,760,008 (99.59%)	7,535,144,309	98.96%	96.98%	49.75%	34,393,458 (69.24%)	9,666,752 (19.46%)	44,060,210 (88.69%)
CTER-3	50,318,732	50,122,976 (99.61%)	7,452,324,140	98.95%	96.97%	49.02%	34,149,363 (70.39%)	8,655,833 (17.84%)	42,805,196 (88.24%)

Notes: Samples: sample name; Raw read: Unprocessed reads directly from the sequencer; Clean read: High-quality reads after QC filtering; Clean Data: Total amount of clean reads; Q20: Bases with accuracy ≥ 99%; Q30: Bases with accuracy ≥ 99.9%; GC: Percentage of guanine and cytosine bases in the clean data; Unique map: Uniquely aligned reads; Mult map: Multi-position aligned reads; Total map: Total aligned reads (Unique + Mult map).

**Table 6:** Significantly enriched signaling pathways in the different treatment groups.

Pathway ID	Pathway	*p*-Value	DEGs
FM-vs.-CAP			
ko04144	Endocytosis	0.000000	75
ko04140	Autophagy	0.000000	44
ko04071	Sphingolipid signaling pathway	0.000003	37
ko04152	AMPK signaling pathway	0.000020	35
ko04910	Insulin signaling pathway	0.000046	36
ko04152	AMPK signaling pathway	0.000020	35
ko04068	FoxO signaling pathway	0.002475	28
ko04915	Estrogen signaling pathway	0.003876	21
ko04625	C-type lectin receptor signaling pathway	0.000035	20
CAP-vs.-CTER			
ko00061	Fatty acid biosynthesis	0.031283	14
ko04145	Phagosome	0.020891	9
ko04210	Apoptosis	0.020891	9
ko04922	Glucagon signaling pathway	0.000477	7
ko04152	AMPK signaling pathway	0.005066	7
ko04152	AMPK signaling pathway	0.005066	7
ko04910	Insulin signaling pathway	0.026043	6
ko00500	Starch and sucrose metabolism	0.002169	5
ko04668	TNF signaling pathway	0.014631	5
ko04973	Carbohydrate digestion and absorption	0.005620	4
ko04622	RIG-I-like receptor signaling pathway	0.008944	4
ko00760	Nicotinate and nicotinamide metabolism	0.018118	3

## Data Availability

The data that support the findings of this study are available upon request from the corresponding author. The data are not publicly available due to privacy or ethical restrictions.
